# Case Report: Metastatic squamous cell carcinoma in the left atrium

**DOI:** 10.3389/fcvm.2024.1500108

**Published:** 2025-01-08

**Authors:** Shuai Luo, Xiaoxue Tian, Ting Xu, Jinjing Wang

**Affiliations:** Department of Pathology, Affiliated Hospital of Zunyi Medical University, Zunyi, Guizhou, China

**Keywords:** heart, metastatic tumours, squamous cell carcinoma, early diagnosis, prognosis

## Abstract

**Background:**

Metastatic tumours are the most common malignant tumours affecting the heart. Cardiac metastatic tumour progression is rapid, with no specific treatments available, and the prognosis is typically poor. Significant challenges remain in the diagnosis and treatment of cardiac metastases.

**Case demonstration:**

A 52-year-old female presented with a history of exertion palpitations lasting over 2 months, worsened by cough and expectoration for 3 days. Colour Doppler echocardiography revealed a hypoechoic mass in the left atrium, which was excised. Postoperative pathology confirmed metastatic squamous cell carcinoma of the left atrium. Six months after surgical removal, the patient remained in good general condition.

**Conclusion:**

Intracardiac metastasis is extremely rare and presents with non-specific clinical symptoms, often leading to oversight by clinicians. Early diagnosis relies on imaging studies, while definitive diagnosis requires pathological examination. Timely detection is crucial to improving patient prognosis.

## Background

Metastatic tumours are the most common type of cardiac malignant tumours, with secondary cardiac tumours occurring at an incidence 20 times higher than primary cardiac tumours (1). These tumours typically originate from lung cancer, breast cancer, malignant melanoma, and lymphoma (2), with rarer origins including prostate cancer (3), renal cell carcinoma (4), and chondrosarcoma (5). Cardiac metastases progress rapidly, lack specific treatments, and are associated with a poor prognosis (6). In cases without an obvious primary tumour, cardiac metastatic lesions are usually advanced at detection. The diagnosis and treatment of cardiac metastases remain significant challenges. We report a case of metastatic squamous cell carcinoma (SCC) of the left atrium to contribute to the understanding of this disease.

## Case demonstration

**Figure 1:**
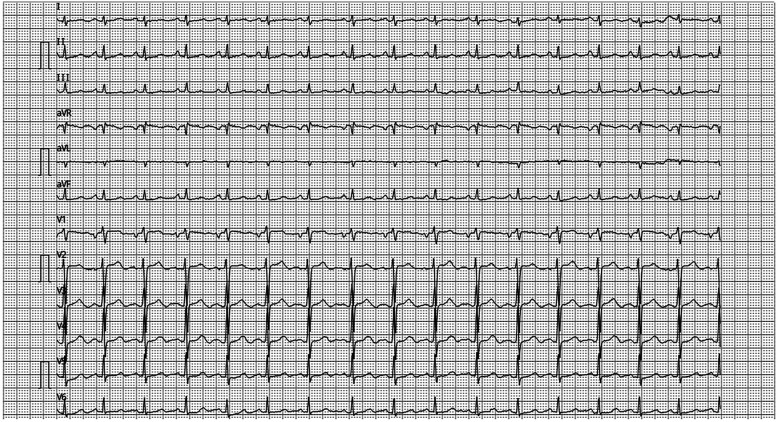
ECG P: V1(+-), Ptf-V1 = −0.04mms, suggesting left atrial overload.

A 52-year-old female presented with exertional palpitations persisting for over 2 months, aggravated by cough and expectoration for the past 3 days. She denied dizziness, headache, nocturnal paroxysmal dyspnoea, orthopnoea, or lower limb oedema. The symptoms had been recurrent for over 2 months, with palpitations worsening during activity 3 days prior, relieved by rest, and accompanied by cough and expectoration. On physical examination, her temperature was 36.5℃, pulse 92 beats/min, respiratory rate 21 beats/min, and blood pressure 112/67 mmHg. Laboratory findings included brain natriuretic peptide 598 pg/ml and ferritin 396.2 μg/L. No bulging or depression was observed in the precordial area, the pulse range and rhythm were normal, and no murmurs were detected on auscultation. A chest radiograph revealed a pear-shaped heart with a slightly increased transverse diameter, indicating mild cardiac enlargement. Electrocardiogram (Figure 1) showed P:V1 (+-), P-terminal force-V1=−0.04 mms, suggesting left atrial overload.

Echocardiography revealed left atrial enlargement and a hypoechoic mass measuring approximately 49 mm × 68 mm in the left atrium, exhibiting minimal systolic and diastolic activity with cardiac motion. Doppler examination showed an anterior mitral flow velocity of approximately 177 cm/s. Mild tricuspid regurgitation and slight aortic regurgitation were observed. Based on tricuspid regurgitation, the estimated pulmonary artery systolic pressure was approximately 64 mmHg. The mitral flow spectrum demonstrated peak E > peak A. A left atrial mass, likely a myxoma, was suspected. Additional findings included increased mitral valve anterior flow velocity, mild tricuspid regurgitation, slight aortic regurgitation, and pulmonary arterial hypertension.

Given the patient's clinical presentation of a left atrial mass with cardiac insufficiency, left atrial tumour resection was performed via open-heart surgery. Intraoperative transoesophageal ultrasound monitoring confirmed a preoperative hypoechoic mass measuring 70 mm × 50 mm in the left atrium. Surgical exploration revealed tumour involvement in the right superior pulmonary vein. The right atrium and atrial septum were opened, revealing that the tumour completely occupied the left atrium without mitral valve involvement. The tumour measured approximately 8 × 7 cm, with a broad base located at the opening of the right pulmonary vein and extending toward the pulmonary hilum. Partial tumour tissue was left residual, and the resected portion was sent for pathological examination. Postoperative transoesophageal echocardiography showed no significant abnormal echoes in the left atrium. However, the echo of the right superior pulmonary vein was heterogeneous, with numerous small veins in the right superior pulmonary vein area entering the left atrium. The main right superior pulmonary vein showed no detectable blood flow.

**Figure 2:**
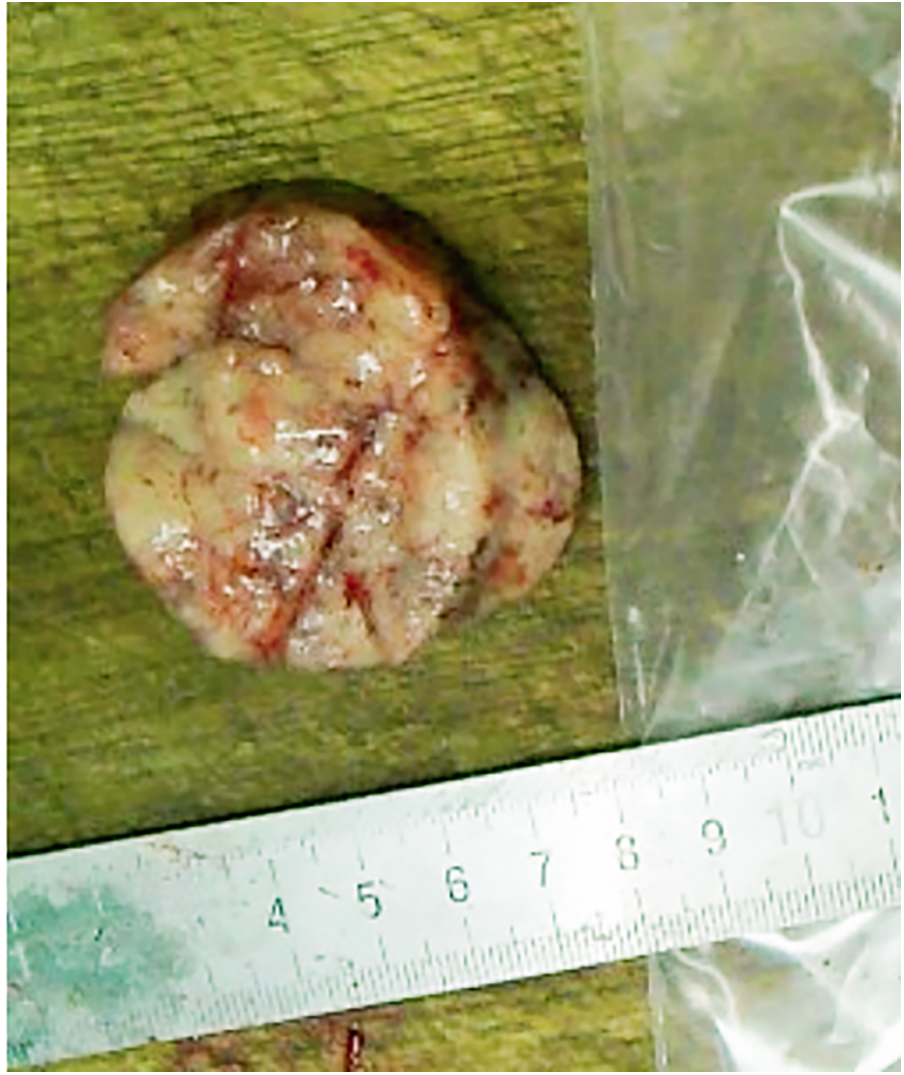
The mass was white cylindrical tissue with white and soft surface and local bleeding.

Gross pathological examination (Figure 2) revealed grey-white, cylindrical tissue measuring 50 × 45 × 15 mm. The cut surface was grey-white with a soft texture and areas of local haemorrhage.

**Figure 3:**
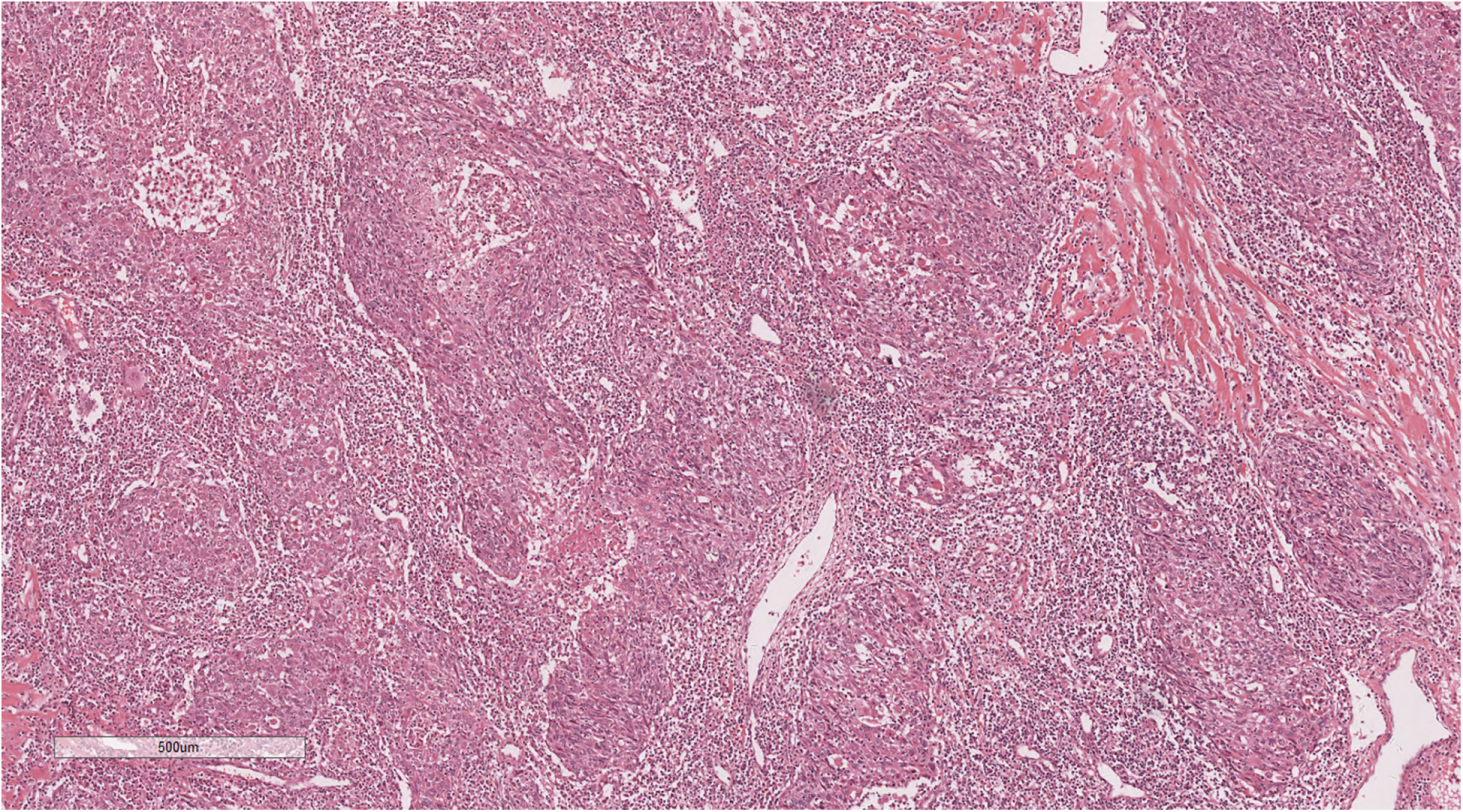
The tumor cells are nested, with a large number of lymphocytes infiltrating between cells and stroma. H&E × 50.

**Figure 4:**
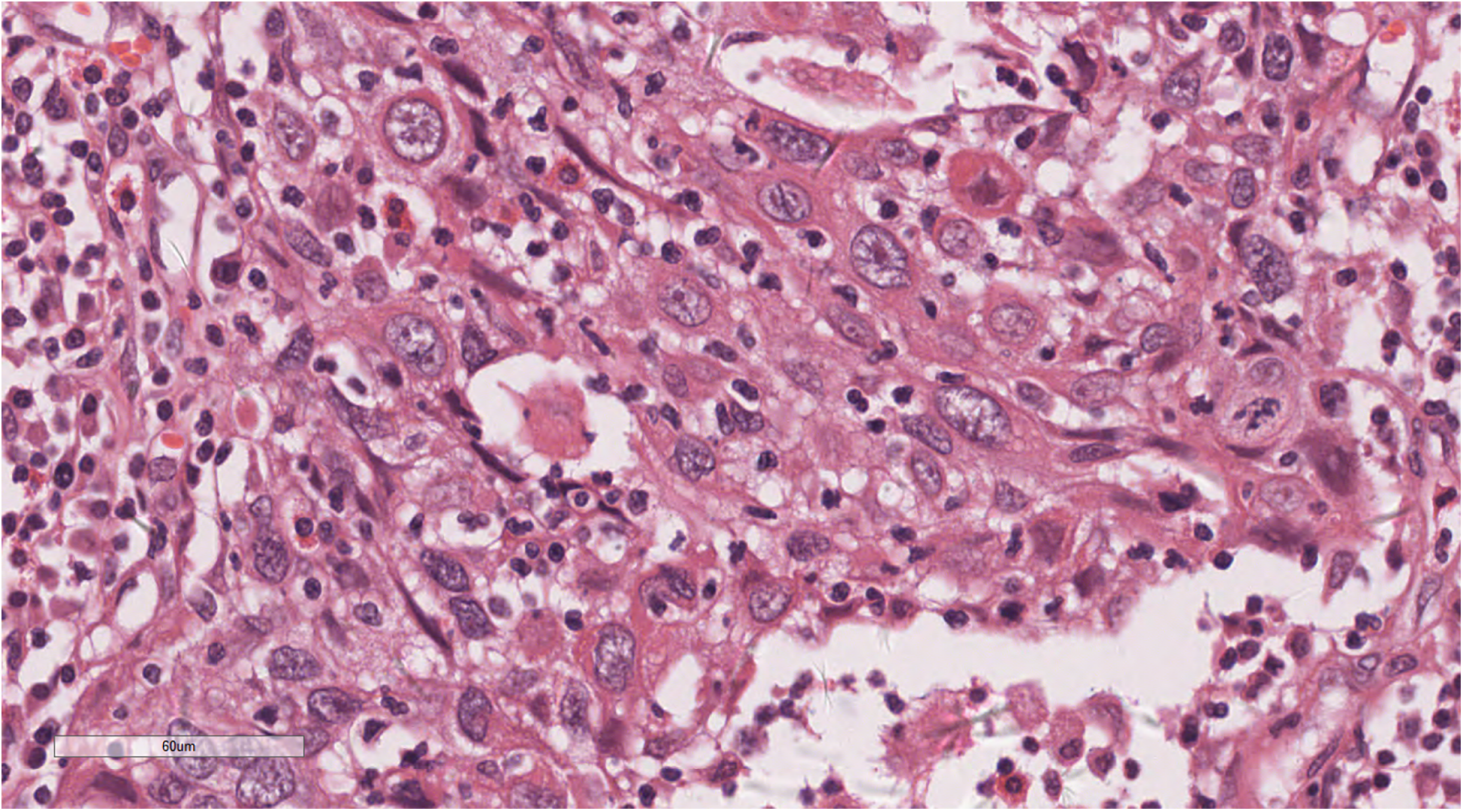
At high magnification, the nuclei of the tumors were round, oval, or elongated, vacuolated, with prominent nucleoli. H&E × 400.

Histopathological examination showed tumours arranged in diffuse sheets or anastomosed, well-defined or irregular islands, with pushing growth at the margins, infiltrating the myocardial tissue. Tumour cells were organised in nests with extensive lymphocyte infiltration between the cells and within the stroma (Figure 3). At high magnification (Figure 4), tumour cell membranes were poorly defined. The nuclei were round, oval, or elongated, with vacuolated and syncytial cells displaying slightly irregular outlines and prominent nucleoli. Some cells exhibited vacuolated or granular chromatin with 1–2 distinct eosinophilic nucleoli. Mitotic figures were also visible. Haemorrhage, necrosis, and lymphocyte infiltration were observed in the centres of the cell nests.

**Figure 5:**
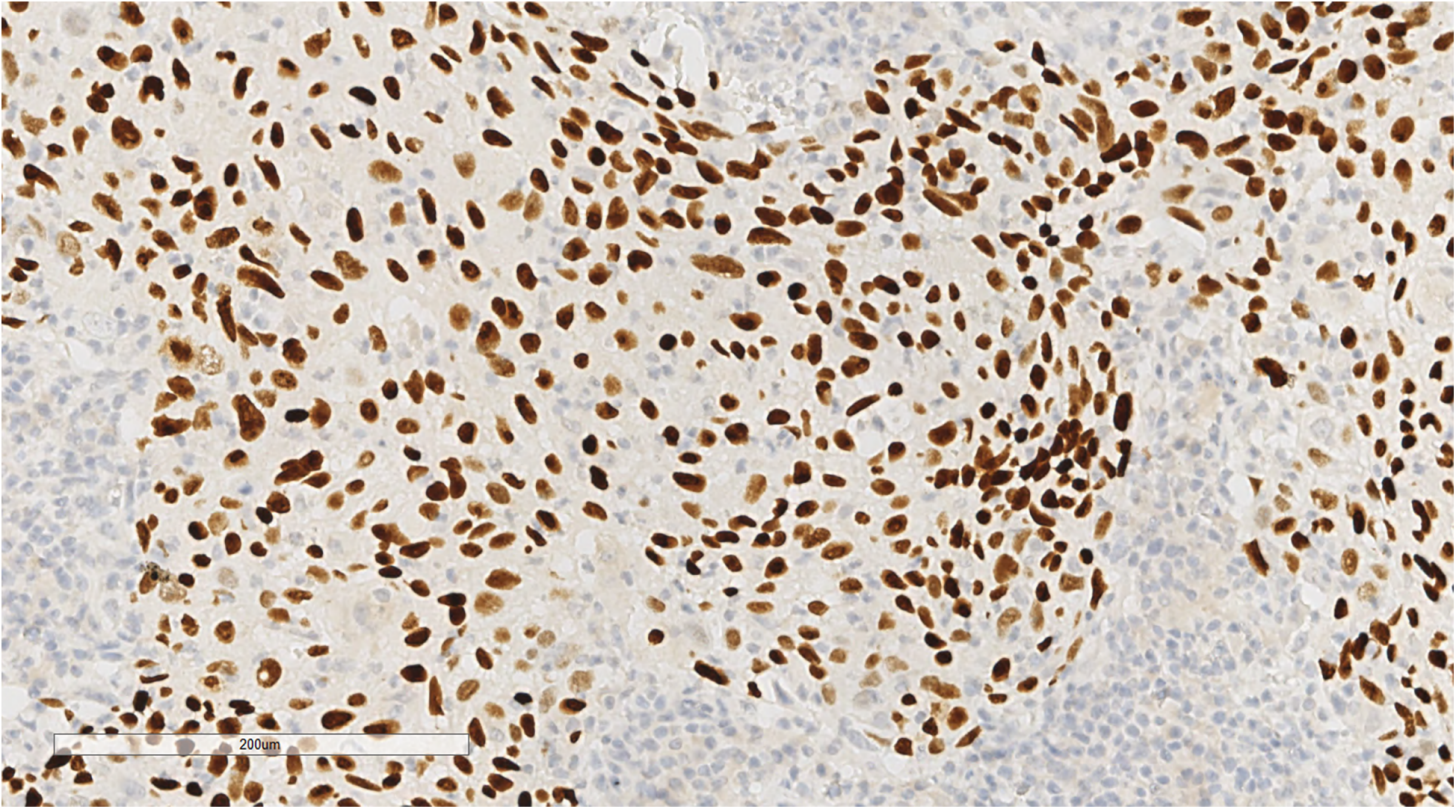
Immunohistochemical staining showed P40(+) tumor cells. EnVision, ×200.

**Figure 6:**
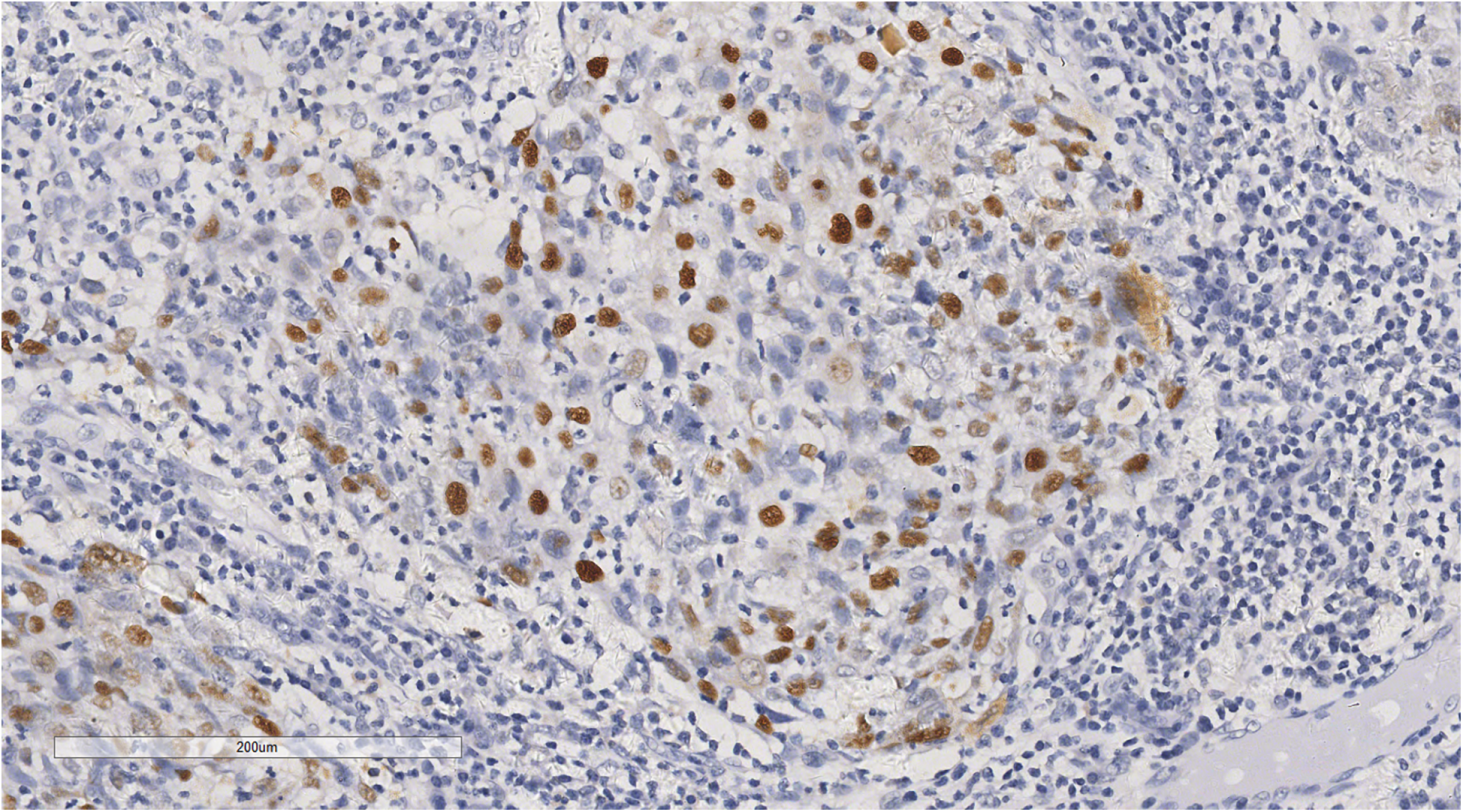
*In situ* hybridization showed that tumor cells were positive for EBER/ISH. ×200.

Immunohistochemical analysis revealed tumour cells positive for cytokeratin (CK) (+++), CK5/6 (+++), P40 (+) (Figure 5), epithelial membrane antigen (weak +), cluster of differentiation (CD) 1a (-), factor VIII (-), S100 (-), CD68 (KP1) (-), CD21 (-), CD23 (-), CD31 (-), CD34 (-), leukocyte common antigen (-), vimentin (-), and Epstein-Barr virus-encoded ribonucleic acid/*in situ* hybridisation (EBER/ISH) (+) (Figure 6).

Based on the histopathological features and immunohistochemical findings, metastatic SCC of the left atrium was diagnosed. The positive EBER/ISH indicated a high likelihood of metastatic nasopharyngeal SCC.

However, the patient declined further nasopharyngeal endoscopy due to personal reasons and opted for conservative management upon discharge. At the 6-month follow-up, the patient's general condition was good, with no evidence of recurrence.

## Discussion

Cardiac metastasis is a rare occurrence with three primary routes: direct extension, haematogenous metastasis, lymphatic metastasis, and intraluminal metastasis via the inferior vena cava or pulmonary vein (7). Metastases in the heart are typically associated with advanced disease and are rarely identified as the initial site of metastasis. This makes it challenging to detect primary lesions or other metastases in cases of cardiac involvement. The utility of pulsed-wave tissue Doppler imaging in assessing the mobility of intracardiac masses has been documented in the literature (8).

Due to the non-specific symptoms of cardiac metastases, clinicians often consider cardiac myxoma as a differential diagnosis before confirming pathology through biopsy. The most common clinical presentation of metastatic cardiac tumours is direct invasion of the cardiac cavity, vena cava, or pulmonary vein. This frequently results in atrioventricular valve obstruction, leading to impaired cardiac pumping function and manifestations of cardiac insufficiency (9).

In the differential diagnosis, metastatic carcinoma of the heart typically exhibits morphological characteristics consistent with epithelial-origin malignant tumours, such as SCC. SCC often demonstrates invasive growth patterns with cell nests appearing as tongue-like, sheet-like, finger-like, or cord-like structures. Well-differentiated SCC might present keratinised pearls within cell nests and intercellular bridge structures. In contrast, adenocarcinoma often displays glandular, cribriform, or related architectural patterns. Myxomas, as benign tumours, are characterised by abundant myxoid stroma and scattered spindle or stellate tumour cells. Tumour cells in myxomas are frequently arranged around blood vessels, with a broad myxoid halo surrounding them. Immunohistochemical staining is essential for differential diagnosis when required. SCC typically stains positive for CK, P40, and P63, while adenocarcinoma exhibits positivity for CK, CK7, thyroid transcription factor 1, and napsin A. Myxoma tumour cells express calretinin, and markers such as actin, CD31, S-100, and vimentin are variably expressed (10).

In this case, the patient had no prior diagnosis of SCC. The cardiac issue was not suspected until symptoms of cardiac insufficiency emerged, prompting further investigations, including echocardiography. The cardiac mass was not detected until auxiliary examinations, such as colour Doppler echocardiography, were performed. Subsequent pathological biopsy confirmed the diagnosis of metastatic SCC, effectively ruling out the possibility of early surgical intervention. Tumour resection in the heart, particularly for malignant tumours, poses significant challenges due to the frequent infiltration of the myocardium. The structural and functional importance of the cardiac muscle wall often limits the feasibility of complete tumour removal, contributing to a higher recurrence rate and a poor prognosis.

Adenocarcinoma is the most common type of metastatic cardiac tumour, accounting for approximately 50% of cases, while SCC is rare, comprising only approximately 7%, and is associated with a poor prognosis (11). In this case, the diagnosis of metastatic SCC was confirmed through pathological biopsy, with the tumour displaying distinct morphological features. Unlike typical cutaneous SCC, the stroma between the tumour cell nests exhibited a pronounced infiltration of lymphocytes, resembling the characteristics of lymphoepithelial carcinoma of the nasopharynx. Given these findings, it is recommended that clinicians consider the possibility of metastatic nasopharyngeal SCC and propose diagnostic procedures such as nasopharyngeal endoscopy and whole-body positron emission tomography-computed tomography to identify the primary tumour. Unfortunately, the patient declined further examination for personal reasons, leaving the primary tumour unidentified. This lack of definitive localisation significantly impacted subsequent treatment planning.

Regarding treatment, there is currently no established standard protocol for managing cardiac metastases. The therapeutic approach primarily focuses on addressing cardiac metastases, which might include surgical resection, local radiotherapy, or systemic chemotherapy (12). For cases where surgical removal is not feasible, symptomatic treatment is employed to alleviate cardiac pressure. The secondary aspect of treatment involves addressing the primary tumour. Literature indicates that while chemotherapy is generally considered the most effective treatment for metastatic cardiac lesions, surgical resection is recommended for left atrial tumours due to the elevated risk of cerebral embolism associated with these masses (13). However, despite advancements in imaging techniques and treatment modalities, the prognosis for cardiac metastases remains poor. This underscores the importance of prioritising palliative care to improve the quality of life for patients with advanced disease stages (14).

## Conclusion

In summary, intracardial metastatic cancer is relatively rare and often presents with non-specific clinical symptoms, making it easily overlooked by clinicians. Early diagnosis relies on imaging studies, while definitive diagnosis is established through pathological examination. For patients with primary malignancies, regular imaging follow-ups are essential for the timely detection of metastatic lesions. Early diagnosis remains crucial for improving patient prognosis.

## Data Availability

The original contributions presented in the study are included in the article/Supplementary Material, further inquiries can be directed to the corresponding author.
